# Use of direct oral anticoagulants in the treatment of left ventricular thrombi: A tertiary center experience and review of the literature

**DOI:** 10.1002/ccr3.1917

**Published:** 2018-11-22

**Authors:** Mohamed Shokr, Abdelrahman Ahmed, Hossam Abubakar, Ziad Sayedahmad, Ahmed Rashed, Luis Afonso, Shaun Cardozo

**Affiliations:** ^1^ Division of Cardiology, Department of Internal Medicine Wayne State University/Detroit Medical Center Detroit Michigan; ^2^ Department of Internal Medicine Wayne State university/Detroit Medical Center Detroit Michigan; ^3^ Division of Interventional Cardiology Wayne State University/Detroit Medical Center Detroit Michigan

**Keywords:** Apixaban, direct oral anticoagulants, DOACs, left ventricular thrombus, Rivaroxaban, thrombolysis

## Abstract

Direct oral anticoagulants can potentially provide a more convenient oral alternative for the management of left ventricular thrombi than Warfarin. These medications do not require frequent monitoring and have less drug‐drug interactions. Randomized controlled trials are needed to further demonstrate their efficacy and safety in this setting.

## 1 BACKGROUND

Left ventricular thrombus (LVT) is a common complication following acute myocardial infarction (AMI) with potential for significant morbidity and mortality. While it complicates different forms of cardiomyopathies and coagulopathies; the most commonly encountered clinical scenario for LVT remains ST‐elevation myocardial infarction (STEMI), particularly anterior STEMI. The incidence of LVT has been declining with the evolvement of the treatment of acute myocardial infarction. It ranges between 2.9% and 15.2% in the era of primary PCI as opposed to 40% and 28% in the prethrombolytic and thrombolytic eras, respectively. The decreased incidence is attributed to earlier reperfusion and aggressive use of antiplatelet and antithrombin therapy.^1^ LVT is associated with a significant risk of systemic embolism. In the prethrombolytic era, ischemic embolic stroke complicated 0.8%‐5.5% of anterior STEMI with LVT being the most likely origin especially with postmortem studies showing that 46% of acute myocardial infarction (MI) patients had LVT.^2^, ^3^ A meta‐analysis of 11 studies concluded that the risk of embolization with echocardiography‐demonstrated LV thrombi after anterior MI is five times higher compared to patients without evidence of thrombi on echocardiography.^4^ Development of LV thrombus is related to Virchow's triad of stasis, endothelial injury, and hypercoagulable state. These components are commonly present following MI, especially anterior MI.^2^ Other factors that increase risk of thrombus formation are advanced age, high levels of C‐reactive protein, presence of apical aneurysm, and low ejection fraction.^5^

Oral anticoagulation with Warfarin is the currently recommended and most evidence‐based therapy. We report our tertiary center experience with the use of Rivaroxaban and Apixaban for treatment of patients with LVT.

## 2 CASES

### 2.1 Case 1

**Figure 1:**
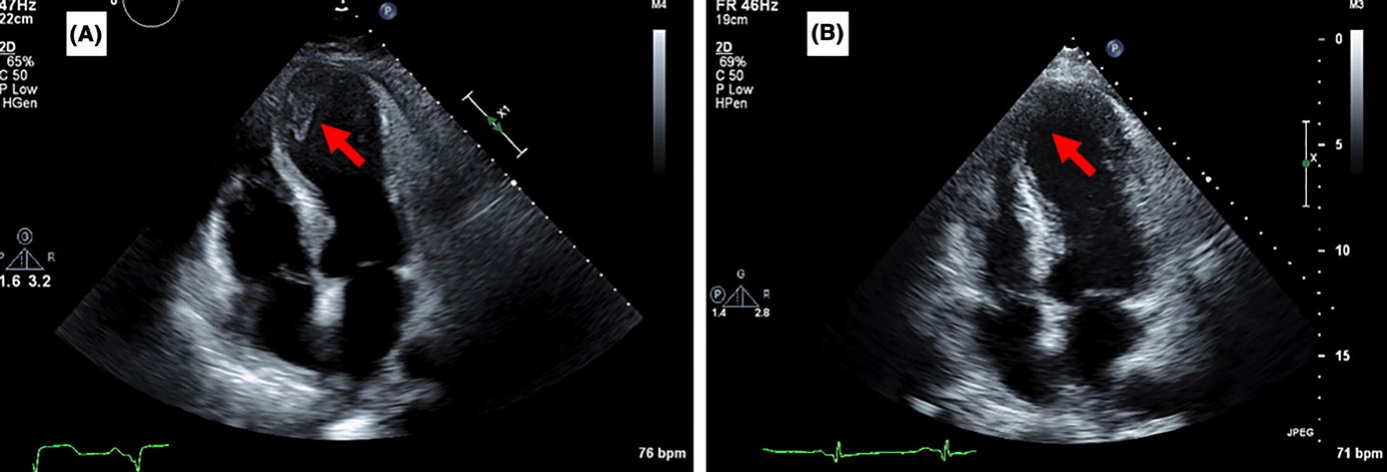
A, Apical four chamber view showing the LV thrombus of case one. B, Resolution of the thrombus after three months of anticoagulation with Rivaroxaban

A 70‐year‐old man with history of hypertension and diabetes mellitus (DM) presented with chest pain due to anterolateral STEMI for which he underwent percutaneous coronary intervention (PCI) of the left circumflex (LCX) with a drug‐eluting stent. There was a chronic total occlusion of the left anterior descending (LAD) as well. A transthoracic echocardiogram (TTE) revealed an ejection fraction (EF) of 10%‐15% with akinetic septum, mid to apical anterior and lateral walls; dyskinetic apex and an echodensity measuring 38 × 18 mm at its greatest dimension suggestive of a thrombus (Figure 1A). HAS BLED score was 1. He was discharged on Aspirin, Clopidogrel, and Rivaroxaban (15 mg daily for 3 weeks then 20 mg daily). A TTE 3 months later revealed resolution of the previously seen LVT and improvement in EF to 35% (Figure 1B).

### 2.2 Case 2

A 63‐year‐old male with history of gout presented with shortness of breath and chest pain. He was found to have new‐onset heart failure due to a completed anterior myocardial infarction for which he underwent PCI to LAD and first diagonal with drug‐eluting stents preceded by rotational atherectomy. TTE showed an EF of 10% with severe akinesia of the apex; anterior, anterolateral, and mid to distal anteroseptal walls with a 12 × 9 mm thin layered mural thrombus. He received intravenous heparin for 7 days while inpatient. HAS BLED score was 1. He was started on Aspirin and Clopidogrel. Due to concerns of compliance with Warfarin, he was discharged on Rivaroxaban 20 mg daily instead. A TTE 4 months later showed resolution of the LVT.

### 2.3 Case 3

A 58‐year‐old man with history of DM, presented with shortness of breath due to new‐onset heart failure secondary to a completed anterior infarction. He underwent PCI to the right coronary artery (RCA) with drug‐eluting stents in the proximal and mid portions. A TTE showed an EF of 10% and a large 18 × 8 mm nonmobile apical thrombus. HAS BLED score was 2 He was discharged on 20 mg of Rivaroxaban in addition to Aspirin and Clopidogrel. A TTE, 3 months later, showed complete resolution of the previously reported thrombus.

### 2.4 Case 4

A 69‐year‐old man with history of ischemic cardiomyopathy presented with acute dyspnea. TTE showed a left ventricular (LV) ejection fraction of 10% with global hypokinesis and a 11 × 13 mm apical LVT. HAS BLED score was 3. He was discharged on Aspirin, Clopidogrel, and Rivaroxaban 20 mg daily which he stopped 40 days later for gastrointestinal bleeding secondary to vascular malformations; however, a follow‐up TTE performed 6 months later showed complete resolution of the LVT.

### 2.5 Case 5

**Figure 2:**
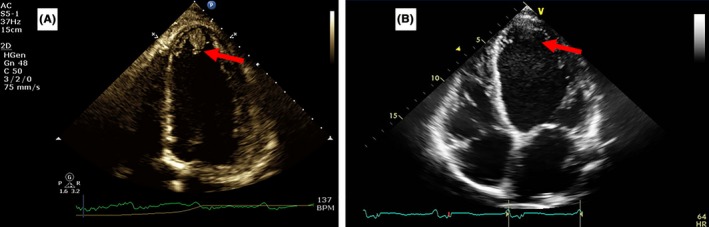
A, Apical four chamber view showing the LV thrombus of case five. B, Resolution of the thrombus after four months of anticoagulation with Apixaban

A 60‐year‐old male with no past medical history presented with shortness of breath and lower extremity edema due to decompensated heart failure following a completed anterior myocardial infarction. TTE showed an EF of 10%‐15%, global hypokinesis and a 19 × 12 mm left ventricular apical thrombus (Figure 2A). HAS BLED score was 0. Due to compliance concerns, he was started on Apixaban 5 mg twice daily following 2 days of intravenous heparin. In addition, he was also discharged on Aspirin and Clopidogrel. He discontinued the medication one month later; however, a follow‐up TTE 4 months from the diagnosis showed complete resolution of the thrombus (Figure 2B).

### 2.6 Case 6

A 28‐year‐old female with history of hypertension presented with worsening shortness of breath, orthopnea, paroxysmal nocturnal dyspnea, and bilateral leg swelling of 1‐week duration. TTE showed global hypokinesia, bilateral ventricular enlargement with an EF of 10%‐15% and a large echodensity in the apical inferolateral aspect of the left ventricle measuring 36 mm × 15 mm consistent with a thrombus. HAS BLED score was 1. Due to compliance concerns, Apixaban 5 mg twice daily was started instead of Warfarin. In addition, he continued to take Aspirin. Complete resolution of the LV thrombus was noted on TTE 10 months later.

### 2.7 Case 7

A 68‐year‐old male with history of nonischemic cardiomyopathy (NICM) and stroke presented with shortness of breath due to decompensated heart failure. TTE revealed an EF of 25%, and an 8 mm pedunculated apical echodensity consistent with a thrombus. HAS BLED score was 3. Due to patient's reluctance to comply with Warfarin, Apixaban 2.5 mg twice daily was started. He also continued Aspirin and Clopidogrel for his previous stroke. Two months later, follow‐up TTE revealed resolution of the thrombus.

### 2.8 Case 8

A 62‐year‐old male with past history of alcohol abuse presented with sub‐sternal chest pain due to an anetroseptal STEMI for which he had PCI to mid‐LAD with a drug‐eluting stent. TTE showed an EF of 15%‐20%, akinesis of the mid‐anterior wall, anteroseptal wall and apex in addition to two echogenic masses attached to the LV apex consistent with LV thrombi with the larger measuring 20 mm × 12 mm. HAS BLED score was 1. Apixaban 5 mg twice daily was started, in addition to Aspirin and Ticagrelor. Seven months later, a follow‐up TTE showed resolution of the thrombi.

## 3 RESULTS

Diagnosis of LVT was confirmed by the interpretation of one cardiologist in each patient. Out of the eight patients reported in this series, seven were males. The mean age was 59.7 years and the mean time until thrombus resolution was 4.8 months. Six patients (75%) had underlying ischemic heart disease and 50% of LVT developed in the setting of acute STEMI. Only three patients were on antiplatelet therapy prior to developing the LVT. All patients continued on DOACs with antiplatelet therapy (87.5% received dual antiplatelet therapy and only one patient received Aspirin alone) after LVT was diagnosed. Despite the increased bleeding risk with combined anticoagulation and dual antiplatelet therapy (Triple therapy),^6^ there was only one case were Rivaroxaban was stopped after 6 weeks due to GI bleeding related to arteriovenous malformations. In patients who received Rivaroxaban, 75% received the dosing for nonvalvular AF (20 mg daily if the creatinine clearance is >50 mL/min) and one patient received the DVT/PE dosing at the provider's discretion. In patients who received Apixaban, nonvalvular AF doses were used.^7^

## 4 DISCUSSION

The diagnosis of left ventricular thrombosis can be established by cardiac imaging with transthoracic echocardiography (TTE) being the preferred screening modality.^8^ The role of the direct oral anticoagulants (DOACs) Apixaban and Rivaroxaban in preventing strokes in atrial fibrillation (AF) patients has been established as reflected in the 2014 guidelines by the American Heart Association/American Stroke Association (AHA/ASA) which recommend either Warfarin or Apixaban for recurrent stroke prevention in AF patients (Class I; Level of Evidence A) with Rivaroxaban designated a Class IIa indication (Level of Evidence B). However, Warfarin, a vitamin K antagonist (VKA) remains the drug of choice in patients with acute MI complicated by LVT (Class I; Level of Evidence C). Low‐molecular‐weight Heparin (LMWH), dabigatran, Rivaroxaban, or Apixaban for 3 months are to be considered as alternatives in this setting when patients are intolerant to VKA therapy because of adverse events that are nonhemorrhagic in nature (Class IIb; Level of Evidence C).^6^, ^9^, ^10^ It is recommended that anticoagulation is initiated as soon as LVT is detected.^11^

Warfarin remains the first‐line choice because of the lack of studies demonstrating the efficacy and safety of DOACs in this cohort of patients. It affects the anticoagulation cascade at multiple steps and carries the advantage of effective reversal in case of bleeding. However, it poses a challenge to both clinicians and patients and its use is declining in favor of DOACs because of its narrow therapeutic index, the need for constant monitoring, dietary restrictions, and extensive drug‐drug interactions.^12^ DOACs are currently used for various indications including prevention of stroke and systemic embolism in nonvalvular atrial fibrillation (AF), venous thromboembolism (VTE) prophylaxis in major orthopedic surgery, treatment of acute VTE and prevention of recurrent VTE.^13^

Recently, several case reports of successful resolution of LV thrombi DOACs were published. To our knowledge, this is the largest case series to date documenting the effectiveness of these medications in treating LVT. Apixaban and Rivaroxaban are two factor Xa (FXa) inhibitors approved for anticoagulation in the United States.^6^ Their rapid onset and offset make bridging therapy unnecessary which is a convenient peri‐procedural option.^14^ In contrast to VKAs, FXa can potentially prevent de novo thrombi and resolve established ones by directly inhibiting free and thrombus‐associated FXa. They reduce the generation of clot‐induced fibrinopeptide A in a similar fashion to hirudin; a potent inhibitor of thrombin.^15^

Rivaroxaban was approved for the treatment of nonvalvular atrial fibrillation, deep venous thrombosis, and pulmonary embolism. It has the added advantage of loosening the plasma fibrin network by modifying it to thicker fibers and larger pores, therefore allowing for greater flow permeation through the clots, rendering them more sensitive to fibrinolytics.^16^ In patients with recent STEMI, combined use of Rivaroxaban and antiplatelets reduced the risk of the composite end point of death from cardiovascular causes, myocardial infarction, or stroke. Despite the increase in major bleeding compared to placebo, fatal bleeding was not significantly increased.^17^ There is increasing evidence questioning the efficacy of VKA to resolve large intracardiac thrombi. Of note, left atrial appendage (LAA) thrombi persisted in 40% of patients undergoing VKA treatment.^18^ Kawano et al reported successful resolution a right atrial thrombus after failure of Warfarin.^19^

Apixaban is characterized by rapid absorption, more than 50% bioavailability, time to peak activity of 2.5‐4 hours, a half‐life of 12 hours (Warfarin is 40 hours) and 25% renal excretion. Moreover, it has predictable pharmacokinetics and can be administered with fixed doses without the need for regular monitoring which makes it a more attractive option for patients and health care providers alike.^12^, ^20^

Many studies support the efficacy of these agents in thrombolysis but no randomized trial specifically examined their use in LVT treatment. The Apixaban Versus Acetylsalicylic acid to Prevent Stroke (AVERROES) trial compared Apixaban versus Aspirin in nonvalvular AF patients who were not suitable candidates for Warfarin treatment and was terminated early due to the strong evidence of Apixaban superiority over Aspirin.^6^ Patients in this trial were followed up for a mean of 1.1 years. Fifty‐one patients in the Apixaban arm developed stroke or systemic embolism compared to 113 in the Aspirin arm (HR 0.45; 95% CI, 0.32‐0.62). Both medications showed comparable risk of major bleeding (1.4%) (1.2%; HR with Apixaban, 1.13; 95% CI, 0.74‐1.75).^6^ Apixaban efficacy and side effects were well studied against Warfarin mainly in patients with atrial fibrillation. The Apixaban for Reduction in Stroke and Other Thromboembolic Events in Atrial Fibrillation (ARISTOTLE) trial was a double‐blind study that randomly assigned patients to receive either Warfarin or Apixaban with a sample size of 18 201 patients. In this study, Apixaban decreased the risk of stroke or systemic embolism by 21%, major bleeding by 31%, and death by 11% compared to Warfarin. It was also associated with lower rates of gastrointestinal bleeding and lower bleeding rates across age‐groups.^21^ Makrides CA demonstrated that short‐duration Rivaroxaban at a low dose (15 mg/d) in combination with a dual antiplatelet therapy (DAPT) was effective for the treatment of left ventricular (LV) thrombus in patients with acute coronary syndromes and drug‐eluting stent implantation, and at low to intermediate bleeding risk.^22^ Resolution of LVT can be confirmed by follow‐up TTE.^2^ The exact time for resolution of the thrombus varies in each patient. A study of 29 patients suffering from LVT after MI demonstrated the resolution of thrombus in 34% of the patients within 6 months of starting treatment.^23^ The 2014 AHA/ASA guidelines recommend for a duration of treatment with oral anticoagulants for 3 months while the European Society of Cardiology 2017 STEMI guidelines recommend a longer duration of treatment up to 6 months.^6^, ^11^ Based on this, a follow‐up TTE every 3 to 6 months is a reasonable recommendation.

Review of the literature demonstrates different doses of DOACs implemented in the management of LVT. Full dose of anticoagulation (5 mg BID for Apixaban and 20 mg daily for Rivaroxaban) and low doses (2.5 mg BID for Apixaban and 15 mg daily for Rivaroxaban) have been used. We have also used different doses in our patients because of the lack of evidence at that time (Table 1). However, in the setting of dual antiplatelet therapy (DAPT), DOACs increase the risk of bleeding.^24^ Therefore, it is recommended that low doses of DOACs are to be used when combined with low dose Aspirin and Clopidogrel.^25^

**Table 1:** Comparison between PubMed‐reported cases between 2012 and 2017 and our 8 cases

Case	Age	Gender M: male F: female	Etiology	Size (mm)	HAS‐BLED	Antiplatelets/Anticoagulants prior to diagnosis	DOAC Dose & duration	Postdiagnosis Antiplatelets/anticoagulants	Complications	Follow‐up echo
Case 1	70	M	STEMI	38 × 18	1	None	Rivaroxaban 15 mg twice daily for 3 wk then 20 mg daily for 3 mo	Aspirin + Clopidogrel	None	3 mo later → resolution
Case 2	63	M	STEMI	12 × 9	1	None	Rivaroxaban 20 mg daily for 4 mo	Heparin (10 d) Aspirin +Clopidogrel	None	4 mo later → resolution
Case 3	58	M	STEMI	18 × 8	2	None	Rivaroxaban 20 mg daily for 3 mo	Heparin (4 d) Aspirin+ Clopidogrel	None	Resolution after 3 mo
Case 4	69	M	Ischemic cardiomyopathy	11 × 13	3	Aspirin + Clopidogrel	Rivaroxaban 20 mg daily for 6 mo	Aspirin + Clopidogrel	After 6 wk → GI bleed (AV malformation) Switched to ASA/Clopidogrel	5 mo later → resolution
Case 5	60	M	Ischemic cardiomyopathy	19 × 12	0	None	Apixaban 5 mg twice a day for 4 mo	Aspirin + Clopidogrel	None	4 mo later → resolution
Case 6	28	F	Nonischemic cardiomyopathy	36 × 15	1	Aspirin	Apixaban 5 mg twice a day for 10 mo	Aspirin	None	10 mo later → resolution
Case 7	68	M	Nonischemic cardiomyopathy	8	3	Aspirin + Clopidogrel	Apixaban 2.5 mg twice a day for 2 mo	Aspirin + Clopidogrel	None	2 mo later → resolution
Case 8	62	M	STEMI	Two masses ,the largest measuring 12 × 20	1	None	Apixaban 5 mg twice a day for 7 mo	Aspirin + Ticagrelor		7 mo later → resolution
Makrides^22^	52	M	STEMI	N/A	1	None	15 mg 3 mo	Aspirin + Clopidogrel	None	Resolution after 1 mo
Makrides^22^	75	M	STEMI	N/A	2	None	15 mg 3 mo	Aspirin + Prasugrel (switched to Clopidogrel)	None	Resolution after 1 mo
Makrides^22^	69	F	STEMI	N/A	2	None	15 mg 3 mo	Aspirin + Ticagrelor (switched to Clopidogrel)	None	Resolution after 2 wk
Padilla Pérez et al^29^	78	M	Dilated cardio‐myopathy	N/A	NA	None	15 mg 1 mo	None	None	1 mo (resolution)
Azizi et al^30^	54	M	STEMI	N/A	1	None	20 mg 1 mo	Aspirin, Prasugrel (switched to Clopidogrel)	None	1 mo (resolution )
Las Casas et al^31^	61	M	Chaga's disease	12.39 × 25.95	NA	Aspirin + Clopidogrel + Warfarin	20 mg 20 mo	None	None	Resolution after 40 d
Nakasuka et al^16^	42	M	Tachy‐cardia induced cardio‐myopathy	20 × 10	NA	None	15 mg 7 d	Heparin+Warfarin (5 d) Switched to Rivaroxaban	None	7 d (resolution)
Maki et al^32^	21	F	Protein C deficiency	26 × 20	NA	None	15 mg 2.8 y	Heparin (6 d) Warfarin (4 d) Argatroban (2 d )	None	Resolution, 24 d from initial treatment
Jamal et al^33^	39	M	STEMI	N/A	NA	None	20 mg 6 wk	Aspirin + Clopidogrel	None	Resolution after 5 wk
Di Nisioet al^34^	52	M	Cancer related thrombosis	30	NA	None	15 mg daily for 3 wk, followed by 20 mg daily for 4 mo	Aspirin + Clopidogrel	None	4 mo (40% size reduction)

Despite the encouraging data that support the use of DOACs in prevention of thromboembolism, they have a few disadvantages. They are more expensive than Warfarin. Apixaban in particular requires adjusting the dose for age, weight, and kidney function. Patients who meet two of the following three criteria (a) age >80; (b) weight less than 60 kg; (c) creatinine level more than 1.5, must receive 2.5 mg two times a day, which is half the usual dose.^12^ The fact that these agents do not require monitoring is an advantage but can make it difficult to assess patients' adherence to treatment.

Major bleeding has been a potential complication noted with the use of anticoagulants. In a meta‐analysis of nine studies involving 51 533 patients, the mean pooled rates of any major bleeding, major GI bleeding, and intracranial hemorrhage (ICH) with Rivaroxaban were 3.32 (95% CI 2.28‐4.25); 2.41, (95% CI 1.25‐3.56) and 0.40 (95% CI 0.17‐0.74) events/100 patient‐years, respectively. Despite significant heterogeneity among those studies, the pooled rates of major bleeding were still low and consistent with those reported in ROCKET AF trial.^26^, ^27^ In our study, only one patient suffered from GI bleeding requiring blood transfusion 2 months after starting Rivaroxaban. The patient had an episode of melena 8 months prior to starting Rivaroxaban and endoscopy at that time showed duodenal arteriovenous malformations, so it is difficult to link this episode entirely to Rivaroxaban use. Rivaroxaban was discontinued, and the patient remained on Aspirin and Clopidogrel and an echo, 4 months later, revealed resolution of the thrombus. We included eight case reports from the literature where patients received DOACs for LVT with no reported GI bleeding. Other advantages for DOACs over Warfarin include fewer drug interactions with some antimicrobial, antifungal, and antiviral medications that involve CYP3A4 and Pgp metabolic pathways.^28^

Our cases add to the body of evidence supporting the efficacy of DOACs in treating LV thrombi. However, this case series is not without its limitations. First, it demonstrated the role of DOACs in only eight patients. Second, the timing of follow‐up echocardiographic examinations was not standardized which makes it difficult to ascertain the exact timing of thrombus resolution in our patients.

Table 1 combines all the reported cases in the literature compared to our case series.

## CONFLICT OF INTEREST

The primary author has no disclosures.

## AUTHOR CONTRIBUTIONS

MS: drafted the first version of the manuscript particularly the Rivaroxaban discussion, acquisition of clinical images. AA: wrote the discussion about Apixaban, reviewed flow of information in the manuscript, analyzed patient data, critically analyzed the manuscript, and responded to reviewers comments. HA: analyzed and collected patient data. ZS: analyzed and collected patient data. AR: involved in critical revision of manuscript for intellectual content. LA: involved in critical revision of manuscript for intellectual content. SC: involved in critical revision of manuscript for intellectual content.
